# Quality of Recovery After Low-Pressure Laparoscopic Donor Nephrectomy Facilitated by Deep Neuromuscular Blockade: A Randomized Controlled Study

**DOI:** 10.1007/s00268-017-4080-x

**Published:** 2017-06-12

**Authors:** Denise M. D. Özdemir-van Brunschot, Gert J. Scheffer, Michel van der Jagt, Hans Langenhuijsen, Albert Dahan, Janneke E. E. A. Mulder, Simone Willems, Luuk B. Hilbrands, Rogier Donders, Cees J. H. M. van Laarhoven, Frank A. d’Ancona, Michiel C. Warlé

**Affiliations:** 1 Department of Surgery, Radboud University Medical Center, Geert Grooteplein-Zuid 10, 6525 GA Nijmegen, The Netherlands; 2 Department of Anesthesiology, Radboudumc, Nijmegen, The Netherlands; 3 Department of Urology, Radboudumc, Nijmegen, The Netherlands; 4 Department of Anesthesiology, Leiden University Medical Centre, Leiden, The Netherlands; 5 Department of Nephrology, Radboudumc, Nijmegen, The Netherlands; 6 Department of HTA, Radboudumc, Nijmegen, The Netherlands

## Abstract

**Background:**

The use of low intra-abdominal pressure (<10 mmHg) reduces postoperative pain scores after laparoscopic surgery.

**Objective:**

To investigate whether low-pressure pneumoperitoneum with deep neuromuscular blockade improves the quality of recovery after laparoscopic donor nephrectomy (LDN).

**Design, setting and participants:**

In a single-center randomized controlled trial, 64 live kidney donors were randomly assigned to 6 or 12 mmHg insufflation pressure. A deep neuromuscular block was used in both groups. Surgical conditions were rated by the five-point Leiden-surgical rating scale (L-SRS), ranging from 5 (optimal) to 1 (extremely poor) conditions. If the L-SRS was insufficient, the pressure was increased stepwise.

**Main outcome measure:**

The primary outcome measure was the overall score on the quality of recovery-40 (QOR-40) questionnaire at postoperative day 1.

**Results:**

The difference in the QOR-40 scores on day 1 between the low- and standard-pressure group was not significant (*p* = .06). Also the overall pain scores and analgesic consumption did not differ. Eight procedures (24%), initially started with low pressure, were converted to a standard pressure (≥10 mmHg). A L-SRS score of 5 was significantly more prevalent in the standard pressure as compared to the low-pressure group at 30 min after insufflation (*p* < .01).

**Conclusions:**

Low-pressure pneumoperitoneum facilitated by deep neuromuscular blockade during LDN does not reduce postoperative pain scores nor improve the quality of recovery in the early postoperative phase. The question whether the use of deep neuromuscular blockade during laparoscopic surgery reduces postoperative pain scores independent of the intra-abdominal pressure should be pursued in future studies.

**Trial registration:**

The trial was registered at clinicaltrial.gov before the start of the trial (NCT02146417).

## Introduction

Laparoscopic donor nephrectomy (LDN) has several advantages over open donor nephrectomy, e.g., shorter length of hospital stay, earlier return to normal physical function and reduced use of analgesics [1]. The use of low intra-abdominal insufflation pressure decreases postoperative pain in laparoscopic cholecystectomy [2, 3], and also evidence exists that postoperative pain is decreased when low-pressure pneumoperitoneum (PNP) is used during LDN [4]. However, the use of low-pressure PNP can impair surgical field visualization [5, 6]. To optimize the quality of the surgical conditions, Madsen et al. [7] used a deep neuromuscular block (NMB) to enhance surgical space, measured as the distance from the sacral promontory to the trocar. Furthermore, Dubois and Staehr-Rye showed that the use of a deep neuromuscular block (NMB) improves surgical conditions during laparoscopic hysterectomy and laparoscopic cholecystectomy, respectively [8, 9].

In this study, we addressed the hypothesis that the use of low-pressure PNP (<10 mmHg) during laparoscopic donor nephrectomy improves the early quality of recovery as compared to the use of standard-pressure PNP (≥10 mmHg). A deep NMB was used to facilitate the use of the low-pressure PNP.

## Methods

### Patients

Sixty-four live kidney donors were recruited between August 2014 and July 2015, and written informed consent was obtained. All adult patients eligible for live kidney donation after multidisciplinary discussion were eligible for this study. Exclusion criteria included: insufficient knowledge of the Dutch language to read the patient information and to fill out the questionnaires, chronic use of analgesics or psychotropic drugs, known or suspect allergy to rocuronium or sugammadex, the presence of neuromuscular disease and the need for rapid sequence induction. The study was approved by the institutional review board, the protocol was published [10] and the study was registered at clinicaltrial.gov (NCT02146417).

### Randomization and blinding

Patients were randomly assigned to two groups: ‘low-pressure PNP,’ defined as 6 mmHg or ‘standard-pressure PNP,’ defined as 12 mmHg. Since the use of deep NMB may influence early postoperative recovery and other outcome parameters, deep NMB was also used in the control group (standard-pressure PNP). The kidney is often more adhesive in men as compared to women. Also the retrieval of a left kidney usually is more time-consuming due to side branches of the left renal vein. To control for these factors, we stratified for gender and side of donor nephrectomy. Block randomization was performed using a computer-generated randomization code.

All surgeons, anesthesiologists and the research team were blinded. All monitors indicating the intra-abdominal pressure were covered during the procedure. After intubation, a nurse opened a sealed envelope containing the allocation of treatment and subsequently installed the intra-abdominal pressure. The same nurse monitored the intra-abdominal pressure and performed adjustments when required. To assess whether the blinding procedure sufficed in keeping the primary surgeon ignorant of the treatment allocation, he was asked to guess at the end of the procedure whether low or standard pressure was used.

### Anesthesia and surgery

All patients received intravenous anesthesia with 1–3 mg/kg propofol and 0.2–0.5 µg/kg sufentanil. Before administration of rocuronium, the TOF-watch (TOF-watch-SX, MSD BV, Oss, the Netherlands) was calibrated. Rocuronium 1 mg/kg was administered, and the patient was intubated. Anesthesia was maintained by continuous infusion of 0.05–0.5 µg/kg/h sufentanil, sevoflurane (1 MAC) and rocuronium 0.3 mg/kg/h. Deep NMB was defined as a post-tetanic count (PTC) of 1–5. All patients received sugammadex 4 mg/kg after surgery. Patients were extubated when the TOF ratio was at least 90%.

All primary surgeons had performed at least 50 laparoscopic donor nephrectomies. First, a Hasson trocar was introduced and the PNP was established. Subsequently three other trocars were placed under direct vision. After opening of Gerota’s fascia, the renal artery, vein and ureter were identified and dissected. When present, the gonadal, suprarenal and/or lumbal vein were clipped and transected. Then, a Pfannenstiel incision was made. The renal artery and vein were transected using an endostapler, and the kidney was extracted using an endobag. The kidney was immediately flushed at the back table.

After surgery, all patients received patient-controlled analgesia (PCA) with intravenous administration of piritramide (bolus 1 mg, lock-out 6 min) and acetaminophen (4000 mg daily). PCA was stopped at day 2 and was replaced by oxycodon. Patients did not receive local anesthetics.

### Evaluation of perioperative conditions

**Fig. 1:**
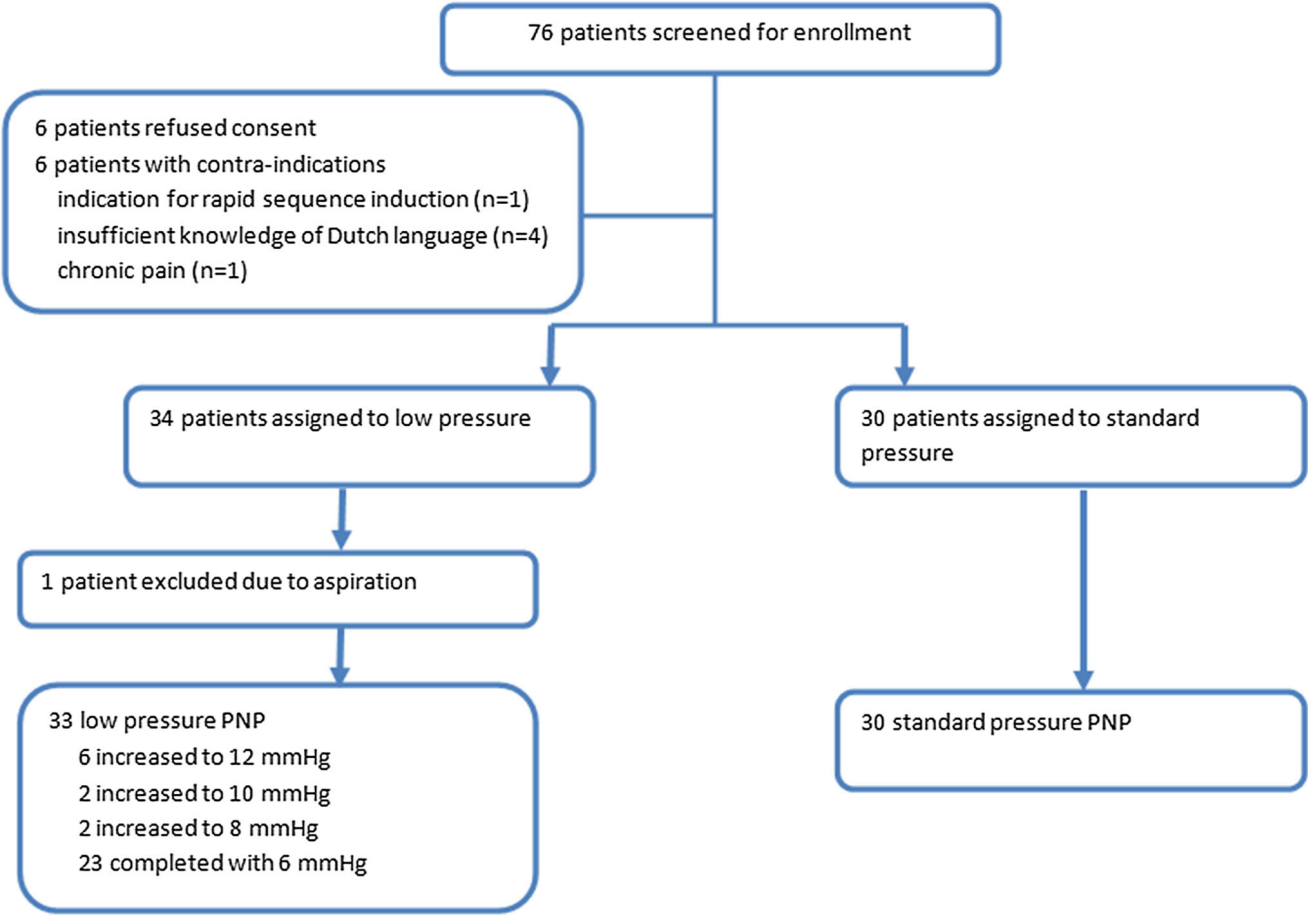
Patient enrollment

During the laparoscopic procedure, surgical conditions were measured after introduction of the trocars and then every 15 min. Surgical conditions were evaluated by means of the surgical rating score (SRS), first described by Martini et al. [11]. The SRS ranged from 1 to 5, extremely poor (1), poor (2), adequate (3), good (4) or optimal (5) depending on the subjective judgment of the primary surgeon. When the overall score was ≤3, intra-abdominal pressure was stepwise increased with 2 mmHg. In case the pressure was already set at 12 mmHg (control group), the nurse was instructed to pretend increasing the pressure stepwise, while keeping the pressure set at 12 mmHg. The study flowchart is shown in Fig. 1.

### Outcome measures

The primary outcome measure was the overall score on the quality of recovery (QoR-40) questionnaire on the first operative day. Secondary outcome measures included: perioperative parameters (PNP) duration, operation time (ORT), first warm ischemia time (WIT1), estimated blood loss (EBL), intra- and postoperative complications and postoperative pain scores. Blood loss was recorded by estimating the amount of blood (ml) in the collection bottle of the suction device after skin closure. Postoperative complications were recorded during the first postoperative days and graded according to the Clavien–Dindo classification. Overall, superficial wound, deep abdominal and referred shoulder pain scores (in rest and upon movement) were recorded as numeric rating scores, using a 11-point Likert scale ranging from 0 to 10 [4]. Superficial wound pain was defined as sharp pain located in the area of the incision(s), deep abdominal pain as a dull and more diffuse pain in the abdomen and referred shoulder pain as pain in the shoulder area.

Also, graft function of the recipient was evaluated. Delayed graft function (DGF) was defined as the need for dialysis in the first postoperative week, excluding when needed for hyperkalemia [12]. Slow graft function (SGF) was defined as serum creatinine >3.0 mg/dl at day 5, without the need for dialysis [13].

### Sample size calculation and data analysis

A ten-point difference in the overall score in the QoR-40 questionnaire on day 1 was considered a minimal clinically relevant difference [14–16]. Based on previous studies, we used a standard deviation (SD) for the QOR-40 score at day one of 14 [14–20]. A sample size of 32 patients per group was required to provide 80% power.

Data were analyzed on an intention-to-treat basis. To control for covariates, i.e., age, gender and side of donor nephrectomy, multivariable logistic regression was used. All analyses were performed using SPSS version 22 (SPSS Inc., Chicago, IL, USA). *p* values <.05 were considered significant.

## Results

### Patient characteristics

*A total of 76 patients were screened for enrollment, six patients refused informed consent and six patients met one of the exclusion criteria. A total of* 34 patients were allocated to the low-pressure PNP group and 30 to the standard-pressure PNP group. For one patient, surgery was canceled due to persistently low oxygen saturation presumably caused by aspiration after induction. According to the protocol, this patient was not replaced. Patient demographics are shown in Table 1, and there were no significant differences in baseline characteristics.

**Table 1:** Patient characteristics

	Low pressure (*n* = 33)	Standard pressure (*n* = 30)	*p* value
Age (year)	54.1 (SD 13.2)	55.9 (SD 10.7)	.57
Male gender	19 (55.9%)	17 (56.7%)	.95
BMI (kg/m^2^)	25.5 (SD 3.2)	26.4 (SD 3.1)	.31
Preoperative serum creatinine (µmol/l)	74.4 (SD 13.1)	75.0 (SD 11.9)	.84

*BMI* body mass index

### Primary outcome measure

Mean QoR-40 score on day 1 was 171.2 (SD 14.5) in the low-pressure group *versus* 165.4 (SD 14.6) in the standard-pressure group (*p* = .12), as shown in Table 2. After correction of age and gender, there was no significant difference in QoR-40 score on day 1 (adjusted *p* = .06). Per-protocol analysis showed a mean QoR-40 score on day 1 of 170.5 (SD 15.6) in the low-pressure group *versus* 166.9 (SD 14.0) in the standard-pressure group (*p* = .35).

**Table 2:** QoR-40 questionnaire

Intention-to-treat	Low pressure (*n* = 33)	Standard pressure (*n* = 30)	*p* value	Adjusted *p* value*
Overall score				
Preoperative	198.9 (SD 1.7)	198.5 (SD 2.6)	.46	.43
Postoperative day 1^#^	171.2 (SD 14.5)	165.4 (SD 14.6)	.12	.06
Postoperative day 2	185.6 (SD 15.3)	179.8 (SD 20.4)	.21	.14
Postoperative day 7	186.2 (SD 12.2)	186.0 (SD 11.5)	.94	.92
Physical comfort				
Preoperative	59.3 (SD 1.4)	59.4 (SD 1.5)	.90	.99
Postoperative day 1	53.2 (SD 5.9)	52.2 (SD 8.8)	.61	.41
Postoperative day 2	53.8 (SD 6.8)	52.0 (SD 12)	.88	.39
Postoperative day 7	55.9 (SD 5.2)	56.6 (SD 3.4)	.57	.56
Emotional status				
Preoperative	49.7 (SD 0.7)	49.3 (SD 1.1)	.11	.10
Postoperative day 1	46.1 (SD 4.0)	46.4 (SD 3.9)	.82	.98
Postoperative day 2	48.4 (SD 5.3)	46.3 (SD 3.9)	.08	**.03**
Postoperative day 7	47.3 (SD 3.6)	46.9 (SD 4.0)	.70	.71
Physical independence				
Preoperative	25.0 (SD 0.2)	24.9 (SD 0.4)	.61	.64
Postoperative day 1	22.7 (SD 7.0)	21.2 (SD 3.7)	.31	.28
Postoperative day 2	21.3 (SD 1.6)	19.7 (SD 3.1)	**.01**	**.00**
Postoperative day 7	22.6 (SD 1.6)	22.4 (SD 1.7)	.67	.64
Support				
Preoperative	30.0 (SD 0.0)	30.0 (SD .2)	.33	.29
Postoperative day 1	21.9 (SD 3.4)	19.9 (SD 2.3)	**.01**	**.01**
Postoperative day 2	29.8 (SD 0.6)	29.5 (SD 1.4)	.21	.16
Postoperative day 7	28.5 (SD 3.3)	29.2 (SD 2.0)	.31	.36
Pain				
Preoperative	34.9 (SD 0.3)	34.9 (SD .4)	.93	.86
Postoperative day 1	31.1 (SD 3.3)	29.5 (SD 4.2)	.12	.08
Postoperative day 2	32.3 (SD 4.9)	32.4 (SD 6.1)	.99	.99
Postoperative day 7	31.9 (SD 2.6)	30.9 (SD 3.4)	.19	.17

Per-protocol	Low pressure ≤10 mmHg^§^ (*n* = 25)	Standard pressure >10 mmHg (*n* = 38)	*p* value	Adjusted *p* value*
Overall score				
Preoperative	198.7 (SD 1.8)	198.7 (SD 2.5)	.95	.94
Postoperative day 1	170.5 (SD 15.6)	166.9 (SD 14.0)	.35	.09
Postoperative day 2	185.6 (SD 11.3)	180.7 (SD 21.7)	.30	.12

Significant *p* values are given in bold

*QoR40* quality of recovery-40 score

^#^Primary study endpoint

* *p* value adjusted for age and gender

^§^For the per-protocol analysis, patients were considered ‘low pressure’ if the intra-abdominal pressure maintained <10 mmHg during the entire procedure

### Secondary outcome measures

Separate analyses of the dimensions of the QoR-40 questionnaire showed that patients allocated to the low-pressure group had significantly higher scores regarding physical support at day 1 (adjusted *p* = .01) and emotional status and physical independence at day 2 (adjusted *p* values are .03 and <.01, respectively), see Table 2. Surgical parameters are shown in Table 3. Mean ORT was 7.8 min longer for low-pressure LDN, which was mainly due to a longer PNP phase. EBL was significantly higher for the low-pressure group, respectively, 48.3 ml (SD 66) versus 22.7 ml (SD 25.4). There were no significant differences in WIT1, conversion to HALDN, or intra-operative complications. With regard to overall pain scores and analgesic consumption, no significant differences were observed between the low- and standard-pressure PNP group, as shown in Table 4. The deep intra-abdominal pain component was significantly lower at postoperative day 2 in patients allocated to the low-pressure group, respectively, 0.8 (SD 1.1) versus 1.8 (SD 2.3).

**Table 3:** Surgical parameters

	Low pressure (*n* = 33)	Standard pressure (*n* = 30)	*p* value
Left kidneys	30 (88.2%)	26 (86.7%)	.85
ORT (min)	109.4 (SD 27.2)	101.6 (SD 23.7)	.23
PNP time (min)	91.6 (SD 30.8)	82.8 (SD 24.9)	.22
Increase in pressure			
8 mmHg	2	0	
10 mmHg	2	0	
12 mmHg	6	0	
Conversion to HALDN	1 (3.0%)	1 (3.3%)	.95
WIT1 (sec)	190.0 (SD 60.8)	199.6 (SD 69.2)	.56
EBL (ml)	48.3 (SD 66.4)	22.7 (SD 25.4)	.**05**

*EBL* estimated blood loss, *HALDN* hand-assisted laparoscopic donor nephrectomy, *ORT* operation time, *PNP* pneumoperitoneum and *WIT1* first warm ischemia time

**Table 4:** Overall and components of pain scores and analgesic consumption

	Low pressure (*n* = 33)	Standard pressure (*n* = 30)	*p* value
Overall maximum pain score^#^			
Postoperative 1 h	4.0 (2.0)	4.1 (2.5)	.84
Postoperative day 1	4.7 (2.3)	4.9 (2.4)	.75
Postoperative day 2	3.7 (2.3)	4.0 (2.4)	.54
Superficial wound component			
Postoperative 1 h	1.8 (2.1)	1.7 (2.2)	.78
Postoperative 1 h (movement)	2.4 (2.4)	2.7 (2.8)	.64
Postoperative day 1	1.1 (1.6)	0.7 (1.4)	.28
Postoperative day 1 (movement)	4.0 (2.5)	3.9 (2.7)	.86
Postoperative day 2	0.6 (1.0)	0.7 (1.3)	.67
Postoperative day 2 (movement)	2.1 (1.7)	2.6 (2.2)	.34
Deep intra-abdominal component			
Postoperative 1 h	2.5 (1.9)	2.3 (2.3)	.75
Postoperative 1 h (movement)	2.5 (2.4)	2.2 (2.3)	.64
Postoperative day 1	1.2 (1.8)	2.1 (2.1)	.09
Postoperative day 1 (movement)	2.7 (2.6)	3.3 (2.6)	.33
Postoperative day 2	0.8 (1.1)	1.8 (2.3)	**.02**
Postoperative day 2 (movement)	2.0 (2.1)	2.7 (2.4)	.18
Referred shoulder component			
Postoperative 1 h	0.3 (1.0)	0.4 (1.5)	.79
Postoperative 1 h (movement)	0.4 (1.2)	0.8 (2.1)	.34
Postoperative day 1	1.3 (1.9)	1.5 (2.3)	.78
Postoperative day 1 (movement)	1.7 (2.4)	1.8 (2.5)	.86
Postoperative day 2	1.6 (1.7)	1.4 (2.2)	.60
Postoperative day 2 (movement)	2.6 (2.2)	1.8 (2.2)	.16
Analgesic medications			
Acetaminophen day 0 (mg)	4000 (0)	4000 (0)	1.0
Acetaminophen day 1	4000 (0)	4000 (0)	1.0
Acetaminophen day 2	3895 (457)	4000 (0)	.27
Piritramide day 0 (mg)	94.2 (101.4)	79.9 (114)	.61
Piritramide day 1	19.3 (18.3)	15.7 (14.2)	.63
Piritramide day 2	0 (0)	0 (0)	1.0
Oxycodon day 0 (mg)	0 (0)	0 (0)	1.0
Oxycodon day 1	12.9 (13.6)	14.1 (6.6)	.79
Oxycodon day 2	5.3 (9.9)	4.8 (5.8)	.33

^#^Maximum score: in rest or after movement including all components of pain (superficial, deep intra-abdominal and referred shoulder pain)

### Surgical conditions and complications

**Fig. 2:**
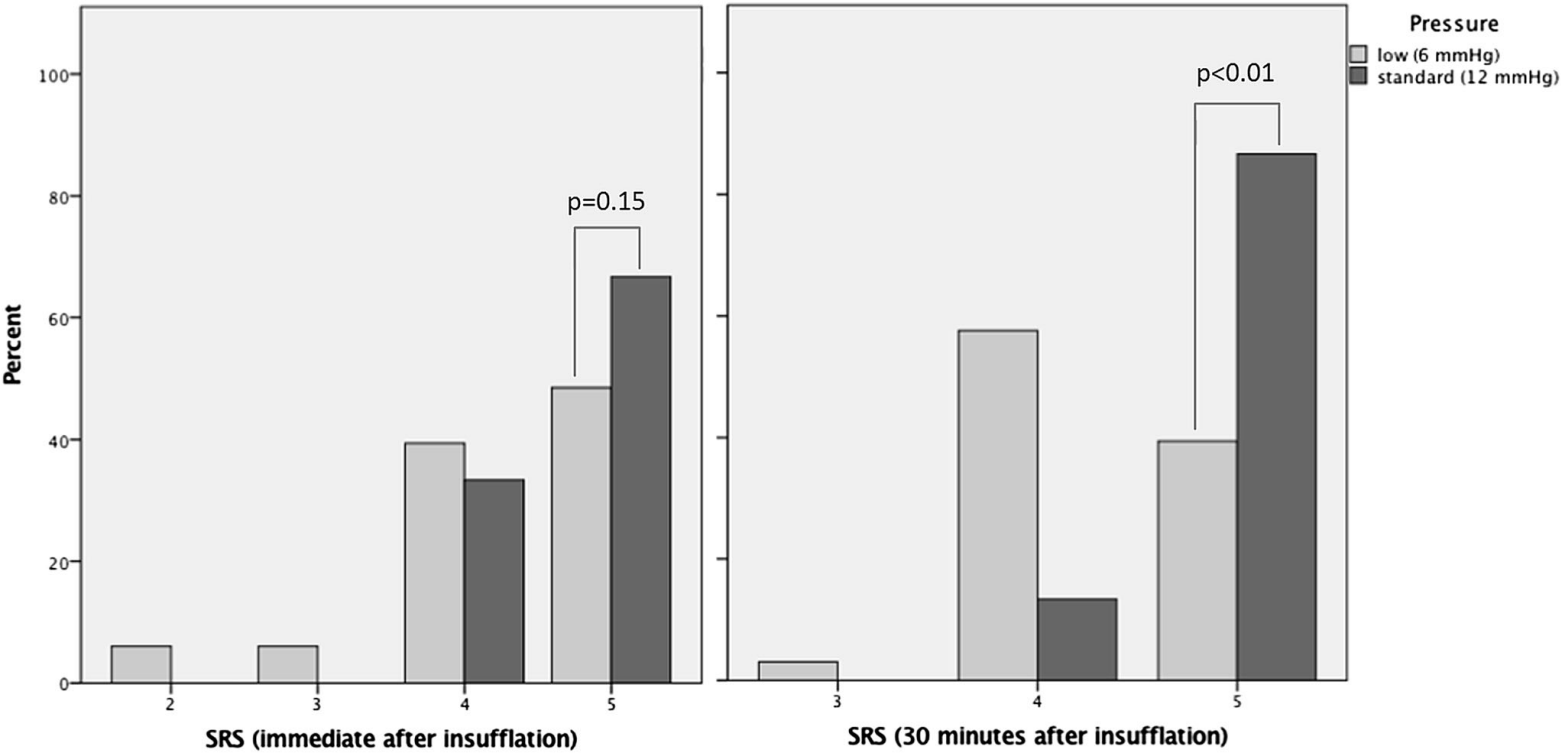
SRS immediate and 30 min after insufflation

During the procedure, it was necessary to increase the intra-abdominal pressure to 8 mmHg in two patients, to 10 mmHg in two patients and to 12 mmHg in six patients. In Fig. 2, it is shown that a SRS score of 5 (optimal conditions) was significantly more prevalent in the standard pressure as compared to the low-pressure group at 30 min after insufflation (*p* < .01).

Intra- and postoperative complications are shown in Table 5. Two splenic lesions occurred. In one patient, a bladder injury occurred after introduction of the endobag. This complication occurred at a time point where the insufflation pressure was increased to 10 mmHg. The lesion was immediately sewed and the urinary catheter remained 5 days in situ where after the patient was discharged without any further consequences. In another patient, the pressure was increased to 20 mmHg to treat persistent venous oozing. This patient was originally allocated to the low-pressure group, but at the time of the bleeding the pressure was already increased to 12 mmHg.

**Table 5:** Length of hospital stay and complications

	Low pressure (*n* = 33)	Standard pressure (*n* = 30)	*p* value
Length of stay (days)	2.8 (SD 1.1)	3.2 (SD 1.1)	.17
Intra-operative complications			
Aspiration	1	0	
Splenic lesion	1	1	
Bladder injury	1	0	
Additional dose of sugammadex	0	1	
Postoperative complications			
Fever of unknown origin^#^	0	2	
Urinary tract infection^§^	1	0	
Pneumonia^§^	0	1	
Gastroparesis^§^	0	1	
Total complications	4	6	.49

^#^Clavien–Dindo grade 1 postoperative complication

^§^Clavien–Dindo grade 2 postoperative complications

For all except two patients, 4 mg/kg sugammadex was sufficient for reversal of deep NMB. In one patient, an additional dose of 2 mg/kg sugammadex was administered. In another, obese patient (100 kg body weight) TOF could not be adequately monitored, possibly due to electrode malpositioning. Therefore, a higher rocuronium dose (12 mg/kg) was administered than actually required.

No significant differences were observed in the length of hospital stay between the low- and standard-pressure group, respectively, 2.8 and 3.2 days (Table 5).

The primary surgeons guessed the initial insufflation pressure at the end of the procedure. In 52 of 63 (82.5%) cases, the surgeon guessed the allocation of treatment correctly.

### Recipient outcome

One recipient died because of an ischemic cerebrovascular accident six weeks after transplantation. With regard to recipient kidney graft function, there were no significant differences in postoperative serum creatinine, or the incidence of DGF or SGF (data not shown).

## Discussion

In this study, patients allocated to the low-pressure PNP group did not show a significantly better quality of recovery at postoperative day 1 which was the primary endpoint of this study. However, patients in the low-pressure group needed less physical support at day 1, and their emotional status and physical independence were significantly better at day 2. The QoR-40 questionnaire is a well-validated, patient-reported outcome measure regarding five dimensions of the quality of recovery after surgery [17]. Nevertheless, the clinical relevance of an improved score in one or more separate dimensions is unclear.

Pain after laparoscopic surgery can be divided into three components: incisional pain, deep intra-abdominal pain and referred shoulder pain [21]. Although the deep intra-abdominal pain score at postoperative day 2 was significantly lower in the low-pressure group, the use of low-pressure PNP did not lead to lower overall pain scores. This is not in accordance with our previous pilot study [4], nor with our recently performed systematic review with meta-analysis comparing pain scores for various laparoscopic procedures [3]. These studies showed significantly lower overall and referred shoulder pain scores in favor of low-pressure PNP. A possible explanation for this discrepancy is that we used a deep NMB in both arms of the study. It has been postulated that a deep NMB more effectively relaxes the abdominal wall musculature as compared to a standard NMB [22]. Therefore, the use of a deep NMB alone (with standard pressure) may reduce pressure-related postoperative pain.

Lindekaer et al. [23] showed that a deep NMB allows a higher intra-abdominal volume with the same intra-abdominal insufflation pressure. To our knowledge, our trial is the first comparing low- versus standard-pressure PNP with the use of deep NMB in both groups. Despite the conversion from low (6 mmHg) to standard pressure (≥10 mmHg) in eight cases (24%), the rating of surgical conditions was significantly better for standard-pressure PNP. Nevertheless, the skin-to-skin operation time was comparable for both groups. More importantly, there was no relevant difference in the intra- and postoperative complication rate between the low- and standard-pressure group. The most important intra-operative complication was an iatrogenic bladder injury in a patient allocated to the low-pressure group. However, this complication occurred at the end of the procedure, while the intra-abdominal pressure was already increased to a standard pressure (10 mmHg) in an early stage. Therefore, it is unlikely to assume that the bladder injury was related to the use of low-pressure PNP.

The main strength of this study is related to its design as a randomized controlled trial. Live kidney donors in general are healthy individuals and therefore provide a highly homogeneous study population. This reduces the risk of confounding bias. To control for factors that may interfere with the outcome measures, we stratified for gender and side of nephrectomy. Although a slight imbalance occurred during block randomization, which resulted in unequal patient numbers in each group, there were no significant differences in baseline characteristics. Another strength of this study is that the study protocol was published beforehand and that we adhered to the study protocol.

A limitation of this study is that eight patients were converted to a standard pressure (≥10 mmHg). Therefore, only 25 patients underwent a ‘true’ low-pressure (<10 mmHg) procedure. This may have blurred the effect on the primary endpoint in the intention-to-treat analysis. The unexpected high rate of conversions to a standard intra-abdominal pressure may be explained by a learning curve for working with lower pressures. Although all patients were operated by experienced laparoscopic surgeons, it cannot be ruled out that less conversions to a standard pressure would have been required if surgeons had more experience with low-pressure conditions during laparoscopy. Our study protocol did not define a per-protocol analysis. However, a post hoc per-protocol analysis also did not reveal a significant difference with regard to the primary outcome measure (Table 2). Another limitation of the study is the fact that the surgeon could not be fully blinded for the use of low-pressure PNP. In this study, the primary surgeons guessed the initial insufflation pressure, and in 82.5% of the cases the surgeon guessed the allocation of treatment correctly. In our view, there is no alternative to overcome this limitation. However, it is important to note that the patients were adequately blinded and that a blinded physician assessed all outcome measures. Although the clinically significant difference of the QoR-40 questionnaire is debatable, several studies with comparable types of surgery have used ten points as a clinically significant difference [14, 16]. After finishing this study, the minimal clinically important difference of the QoR-40 questionnaire was found to be 6.3 in a study by Myles et al. [24]. In our study, the differences in the QoR-40 score at postoperative day 1 between low- and standard-pressure group after intention-to-treat and per-protocol analyses were 5.8 and 3.6, respectively. As these differences are smaller than the minimal clinically important difference, it seems unlikely that a larger sample size would lead to different conclusions.

In conclusion, the use of low-pressure pneumoperitoneum with deep NMB did not reduce postoperative pain scores or improve the overall quality of recovery after LDN. As a deep neuromuscular block was also applied in patients allocated to the standard-pressure group, the questions arise whether deep NMB reduces intra-abdominal pressure-related pain independent of the intra-abdominal pressure. This issue should be addressed in future studies.

## Abbreviations

CRR2: Creatinine reduction ratio on day 2
DGF: Delayed graft function
EBL: Estimated blood loss
LDN: Laparoscopic donor nephrectomy
ORT: Operation time
NMB: Deep neuromuscular block
PNP: Pneumoperitoneum
PTC: Post-tetanic count
SGF: Slow graft function
TOF: Train-of-four
WIT1: First warm ischemia time
